# Pilot Validation Study: Canadian Global Rating Scale for Colonoscopy Services

**DOI:** 10.1155/2016/6982739

**Published:** 2016-10-20

**Authors:** Stéphanie Carpentier, Nour Sharara, Alan N. Barkun, Sara El Ouali, Myriam Martel, Maida J. Sewitch

**Affiliations:** ^1^Division of Gastroenterology, McGill University and McGill University Health Center, Montreal, QC, Canada; ^2^Division of Clinical Epidemiology, Research Institute of the McGill University Health Centre, Montreal, QC, Canada

## Abstract

*Background*. The United Kingdom Global Rating Scale (GRS-UK) measures unit-level quality metrics processes in digestive endoscopy. We evaluated the psychometric properties of its Canadian version (GRS-C), endorsed by the Canadian Association of Gastroenterology (CAG).* Methods*. Prospective data collection at three Canadian endoscopy units assessed GRS-C validity, reliability, and responsiveness to change according to responses provided by physicians, endoscopy nurses, and administrative personnel. These responses were compared to national CAG endoscopic quality guidelines and GRS-UK statements.* Results*. Most respondents identified the overarching theme each GRS-C item targeted, confirming face validity. Content validity was suggested as 18 out of 23 key CAG endoscopic quality indicators (78%, 95% CI: 56–93%) were addressed in the GRS-C; statements not included pertained to educational programs and competency monitoring. Concordance ranged 75–100% comparing GRS-C and GRS-UK ratings. Test-retest reliability Kappa scores ranged 0.60–0.83, while responsiveness to change scores at 6 months after intervention implementations were greater (*P* < 0.001) in two out of three units.* Conclusion*. The GRS-C exhibits satisfactory metrics, supporting its use in a national quality initiative aimed at improving processes in endoscopy units. Data collection from more units and linking to actual patient outcomes are required to ensure that GRS-C implementation facilitates improved patient care.

## 1. Introduction

Since 2010, all Canadian provinces have either announced or started implementing organized CRC screening. The increase in colonoscopy volume coupled with the variability in colonoscopy service quality across sites has ignited a movement for quality assurance [1–4]. Current CRC screening guidelines emphasize quality in colonoscopy, and the Canadian Association of Gastroenterology (CAG) began a quality program in endoscopy in 2012-2013 [5]. Central to the CAG's program is the Global Rating Scale (GRS), an endoscopy quality improvement tool that was developed in 2005 in the United Kingdom (UK). This 12-item GRS-UK questionnaire was developed following meetings with endoscopy staff [6, 7] who were instructed to consider areas that would be important for a patient undergoing endoscopy. The GRS program offers endoscopy facilities the ability to evaluate the quality of their services according to a routine schedule and to then evaluate the effects of targeted quality improvement interventions. The GRS-UK has proven effective in improving endoscopy services, and while no formal validation studies for GRS-UK have been performed, some groups in the UK, Netherlands, and Scotland have attempted to validate patient involvement in the GRS [8, 9]. Experts in Canada were concerned that the tool may not be relevant to the Canadian public or the Canadian health care system, because the quality items were generated by health professionals in the UK who work in and whose patients are served by a different healthcare system. Thus, a Canadianized version of the GRS (GRS-C) was created [10]. As of July 2015, 109 sites participate in this national GRS-C quality initiative, as part of a concerted nation-wide quality initiative.

Similar to the UK version of the GRS, the GRS-C measures two domains: clinical quality and quality of patient experience. The clinical quality domain includes six items: appropriateness, information/consent, safety, comfort, quality of the procedure, and communicating results. The quality of patient experience domain also includes six items: equality, timeliness, booking and choice, privacy and dignity, aftercare, and ability to provide feedback. Each of these items, in turn, includes a series of graduated statements, and based on the response to these graduated statements, the endoscopy suite is scored on a scale that ranges from A to D (A being the highest and D being the lowest scores).

We sought to examine the psychometric properties of the GRS-C: specifically, validity (face, content, and construct), test-retest reliability, and responsiveness to change.

## 2. Methods

### 2.1. Participating Sites

A multisite prospective cohort study was undertaken in endoscopy facilities at the Royal Victoria and Montreal General Hospitals (of the McGill University Health Centre) in Montreal and the Queen Elizabeth II Health Sciences Centre in Halifax, Nova Scotia (see Table 1 for characteristics of participating sites).

### 2.2. Study Population

A staff committee at each site was convened to complete the GRS-C that comprised an endoscopist, nurse, administrative staff, endoscopic technical assistant, and a representative from the management team. The members of the committee remained constant throughout the study and were experienced in completing the GRS, which had already been adopted at the time of study inception. These staff committees completed all questionnaires on psychometric testing except for face validity, in which a separate staff committee was recruited to complete face validity questionnaires. Recruitment of the face validity group was based on lack of familiarity with the GRS tool and availability.

### 2.3. Validity and Reliability Testing

#### 2.3.1. Face Validity

The statements from each domain were isolated without accompanying descriptive information. We then asked the participants to write, in one sentence or less, what overarching theme they thought the statements intended to measure.

#### 2.3.2. Content Validity

We systematically examined GRS-C items to ensure they included accepted key elements of a quality colonoscopy experience [4]. Two members of the research team (NS and SC) compared the GRS-C to the content of the “Canadian Association of Gastroenterology (CAG) Consensus guidelines on safety and quality indicators in endoscopy” [1], and disagreements were resolved through an independent third party. We also examined the percentage of statements in common for each item in the GRS-C and the reference GRS-UK.

#### 2.3.3. Construct Validity

We looked at the degree to which the GRS-C measured the quality aspects being investigated in two ways. First, at all sites, the staff committee completed the GRS-C and the GRS-UK on the same day, and scores of the GRS-C items were compared to those of the reference GRS-UK. Second, at one site, we looked at patient outcomes data that related to GRS-C statements. We compared responses of repeat GRS-C administration against actual patient experience data collected simultaneously. Patient experience data was extracted from a “patient satisfaction survey” administered to every 5 colonoscopy patients until a total of 500 surveys were administered. Surveys were given to every 5th patient by units nursing staff, to be completed at home and returned anonymously in provided prestamped envelopes. 272 were returned for a response rate of 54% (sample patient satisfaction survey available on GRS-C website) [11].

#### 2.3.4. Test-Retest Reliability

For* reliability* testing, we examined whether responses to the GRS-C were consistent when administered under consistent conditions. Staff committee members completed the GRS-C at time zero and again two weeks later, without changing any aspect of endoscopic services delivery. The staff committees were blinded to the purpose of the retest.

#### 2.3.5. Responsiveness to Change

At the time of GRS-C completion, deficiencies were identified and one or more action plans were created locally to address site-specific deficiencies. Six months following the implementation of the action plan, the GRS-C was completed by the staff committees. GRS-C scores before and following planned implementation of these changes were compared.

#### 2.3.6. Statistical Analysis

Face validity was analyzed qualitatively by comparing each of the staff committee member's response to the known domain theme. Discrepancies were noted.

Content validity was evaluated as the proportion of the CAG Consensus guidelines on safety and quality indicators in endoscopy that were represented in the GRS-C statements and the percent overlap with the reference GRS-UK. Construct validity was assessed by comparing the overall grade (A-D) for the 12-item scores of the GRS-C with those of the reference GRS-UK when both were administered at the same time. Comparisons of selected endoscopy unit outcomes corresponding to distinct GRS-C statements were carried out. Descriptive statistics included proportions with their corresponding 95% confidence intervals.

Reliability was assessed using Kappa scores calculated on the 12-item scores of the GRS-C administered at baseline and 2 weeks. Responsiveness to change was assessed using the McNemar chi-square test for paired data calculated, comparing the individual 12-item ratings of the GRS-C administered at baseline and 6 months following improvement interventions.

## 3. Results

### 3.1. Face Validity

As outlined in Table 2, for the twelve groups of statements, the majority of participants correctly identified the intended overarching theme.

### 3.2. Content Validity

Of the 23 key quality indicators identified in the CAG Consensus guidelines on safety and quality indicators in endoscopy, 18/23 (78%, 95% CI (58; 90)), were addressed in the GRS-C. The GRS-C did not evaluate education and monitoring of trainees within the endoscopy suite or education of staff nor did it evaluate criteria for maintaining endoscopist privileges (Table 3).

When the content of the GRS-C and the GRS-UK was compared, 9 of the 12 GRS-C items had greater than/equal to 70% content overlap in statement content. Appropriateness, communicating results, and equality of access were the 3 items that fell below this level (Table 4).

### 3.3. Construct Validity

For site 1, 75% (95% CI; 47%–91%) of the GRS-C item ratings were the same as obtained in the corresponding reference GRS-UK items. For sites 2 and 3, 100% (95% CI; 76%–100%) and 92% (95% CI; 65%–99%), respectively, were the same as GRS-UK.

The response to GRS-C statements and corresponding outcome data are detailed in Table 5.

### 3.4. Test-Retest Reliability

Test-retest reliability ranged from .65 to .83. At site 1, it was “almost perfect agreement” (kappa = 0.83; 95% CI 0.73; 0.93), while for sites 2 and 3 agreement was “substantial” (kappa = 0.81; 95% CI 0.70; 0.92) (kappa = 0.65; 95% CI 0.51; 0.80).

### 3.5. Responsiveness to Change

Table 6 lists the separate initiatives that were attempted or carried out between the baseline and 6-month follow-up GRS administrations. At site 1, no differences were found comparing pre- and post-GRS responses; however, significant differences were noted for sites 2 and 3 (*P* < .001).

Various improvement initiatives were undertaken in the 6-month interval between baseline and the second iteration. Site 1 created a patient information pamphlet on colonoscopy and increased the frequency of quality assurance reviews (endoscopist, adverse events, and general unit review). Site 2 created and implemented a patient satisfaction survey, translated patient related materials into French, and improved tracking of cancellation rates. Site 3 created a patient satisfaction survey, increased review of direct to procedure guidelines, and implemented reliable electronic distribution of reports to referring physicians.

## 4. Discussion

In this multisite study, we tested the validity and reliability of the GRS-C that is increasingly used in Canada to improve the quality of endoscopy services [12].

In assessing face validity, participants were able to correctly interpret the items of the GRS-C, despite a lack of knowledge of the tool. Three participants interpreted the “consent process” as reflecting “patient satisfaction,” perhaps because many of the statements appear to be centered around the patient's satisfaction with how consent is obtained. The extent to which the patient opinion is captured in the GRS instrument is unclear as the GRS mainly focuses on processes in the endoscopy service delivery.

We found substantial overlap between the GRS-C and the CAG Consensus guidelines on safety and quality indicators in endoscopy. The presence and monitoring of educational programs, both for staff themselves and for GI trainees, are not explicitly addressed by the GRS-C. However, implicit in the “quality of the procedure” domain is the understanding that, to favor self-improvement, endoscopists will have to embark on continuing professional development activities. National consensus on what constitutes an effective training program would need to be more precisely defined for all endoscopy units before specific educational initiatives can be agreed upon.

Similarly, before statements regarding maintenance and revocation of privileges can be made, “maintenance of colonoscopy certification” standards may have to be agreed upon by all participating professional societies. The CAG is leading such an initiative, beginning with the hands-on Skills Enhancement in Endoscopy courses now available across the country [13].

In comparison to the GRS-UK, 10 out of the 12 GRS-C items showed greater than or equal to 70% overlap in content, while three fell below this level. The main difference between the GRS-UK and GRS-C for item 6 involves the emphasis on enforcing standardized electronic reporting. This is emphasized in the CAG Consensus guidelines, and so we believe these statements are important additions to this item. The GRS-UK includes standardized timeframe within which results of pathology reports should be acted upon once received. The GRS-C does not include this, potentially because timelines for turnaround for pathology reports themselves are currently variable from institution to institution across Canadian provinces.

There are several differences between the GRS-UK and the GRS-C for Item 7 (equality of access). The GRS-UK includes a statement discouraging family and friends from acting as interpreters. This may be an important ethical addition to the standards set out in the GRS-C. The GRS-UK also sets out a standard that communication method for all groups should be clearly and individually outlined in a policy statement. This level of policy detail is not demanded in the GRS-C. The GRS-C however does require that action plans and results thereof be regularly reviewed, such as part of a planned follow-up to annual patient surveys that will ultimately lead to a patient centred standard of care for diversity.

Undoubtedly, it would be helpful to align all items of the GRS-C with auditable outcomes to more explicitly measure the construct validity of the GRS-C. Interestingly, in statement 1.11 (availability of patient information), although the committee responded that information sheets were provided, only two-thirds of patients surveyed reported having received these. This highlights differences between the actual patient experience and the perceived patient experience as measured by the completing committee. Ongoing feedback from patients to the responsible unit managers is important to ensure that policies are consistently implemented.

For reliability testing, GRS-C completion at baseline and at 2 weeks was chosen in order to study interpretation of items and reliability of responses without having confounding from differences brought about by interval systemic change. The GRS-C proved reliable in this study; in fact reliability was almost perfect at one site and was substantial in the other two. In reviewing feedback provided by the completing committees, although filled out by the same people in all iterations, variability in interpretation of the items can explain the few inconsistent responses. For example, in statement 3.4 (unacceptable comfort levels prompt a review during the procedure…) one centre identified that most, but not all, endoscopists review indications, technique, and sedation levels when unacceptable comfort levels are reached. The question was initially interpreted as “yes” because most physicians did this, but 2 weeks later the group answered “no” as they focused on the minority of endoscopists who do not adhere to this practice. Similarly, for item 5.2 (“Surveillance and screening endoscopy is booked according to established guidelines”) certain physicians do not routinely follow these guidelines, but since the “majority” do, two sites answered “yes” to the question. Members of the staff committee were unsure if this was the “right” way to respond. This finding speaks to the need for more effective management and more uniform policy statements across all endoscopy facilities and their staff.

A partial solution to the issue of interpretation variability may be to ensure, as much as possible, that the same group completes the GRS at each cycle. Indeed, this may increase consensus on interpretation and increase reliability over time. Furthermore, the current study looked at iterations only 6 months apart.

Two out of the three sites demonstrated significant responsiveness to change at 6 months, after action plans have been implemented. Both centres had introduced the GRS-C at study inception and it may be that significant improvements could be made as a result of only a few key actions. For example, the creation and distribution of a patient satisfaction survey addressing several GRS statements improved the performance of the unit greatly. It may be that as a unit improves in service delivery, it requires more detailed interventions to continue achieving improvements in GRS-C ratings. Indeed, site 1 commented that many of the detailed action plans put in place at week 2 were still in progress 6 months later. Several interventions were planned, but responders commented at 6 months that time frames were difficult to estimate without input from all unit staff or upper management in charge of resource allocation. It would be interesting to continue this study at 6, 12, and 18 months and assess responsiveness to change within these more extended time frames. Other than the short 6-month follow-up period, a limitation of this pilot study was the relatively small number of participating sites, as well as the limited availability of outcomes data to assess construct validity (available from 1 site only).

## 5. Conclusion

In conclusion, this pilot study provided support for use of the GRS-C. Our findings showed satisfactory face validity, content validity, construct validity, and reliability. Responsiveness to change was demonstrated at the two endoscopy units with less experience with the GRS-C. Further studies are needed to confirm these findings.

## Figures and Tables

**Table 1:** Description of the hospital centres included in the study.

Characteristics	Centre 1	Centre 2	Centre 3
Total number of colonoscopists (*n*)	18	10	14
Gastroenterologists (*n*)	12	7	12
Number of endoscopy rooms	5	5	4
Number of colonoscopies/year (2012-2013)	3526	6860	5662

**(a):** 

GRS-C item	Interpretation of “clinical quality” item: *what aspects of colonoscopy did subjects feel were being addressed? N = 5*	Item intended by rating scale designers
1	Patient satisfaction (3) Informed consent (2)	Consent process

2	Safety and accountability (3) Quality control (2)	Safety

3	Comfort (4), sedation (1)	Comfort

4	Auditable outcomes (1) Quality indicators of care (3)^*∗*^	Quality of the procedure

5	Adherence to guidelines (4) Triage priorities (1)	Appropriateness

6	If/how reports are submitted to referrer, documentation of procedure, reports (5)	Communicating results to referrer

^*∗*^One participant did not respond to the question regarding domain 4.

**(b):** 

GRS-C item	Interpretation of “quality of patient experience” item: *what aspects of colonoscopy did subjects feel were being addressed?*	Domain intended by rating scale designers
7	Access to services (1) Equality of access as it relates to patient's communication (1) Communication with the patient (3)	Equality of access

8	Triage process and wait times (2) Triage process (1) Wait times (2)	Timeliness

9	Scheduling of appointments (3) Appointment scheduling process (2)	Booking and choice

10	Recovery (1) Patient dignity (1) Privacy of patients (1) Maintaining patient's privacy and dignity (1) Patient's privacy and care before and after an endoscopy (1)	Privacy and dignity

11	Results to patients (1) Continuity of care after endoscopy (1) Follow-up and safety of patient (1) Discharge/postprocedure information (1) How to book a follow-up with a physician after a procedure (1)	Aftercare

12	Patients and staff: better communication of complaints and feedback (3) Maintaining quality of care (1) Actions taken when it comes to feedback, surveys, comments or complaints (1)	Ability to provide feedback

**Table 3:** CAG Consensus guidelines on safety and quality indicators in endoscopy.

Statement	Addressed in GRS-C yes/no? (SC)
(1) Informed consent	*Yes*
(2) Adoption of universal standards	*Yes*
(3) Appropriateness	*Yes*
(4) Technical and personnel resources	*Yes*
(5) Preprocedure information	*Yes *
(6) Intraprocedural policies to be implemented	*Yes* (refers to CAG auditable outcomes but does not specifically ask about each)
(7) Adherence to appropriate discharge policies	*Yes* (addressed but does not suggest need for documentation of standard discharge readiness score)
(8) Follow-up policy in place	*Yes*
(9) Provision of written discharge information	*Yes* (NB: does not include discussion of worrisome sx to watch for)
(10) Existence of formal QI program at facility	*Yes *
(11) Existence of a formal quality review committee	*Yes*
(12) Regular review of quality indicators with action plan	*Yes *
(13) Regular review of safety indicators with action plan	*Yes *
(14) Presence of education programs for staff	*No*
(15) Appropriate monitoring and evaluation of trainees	*No*
(16) Ensured competency of all trainees and staff (required documentation of procedures performed, direct observation)	*No*
(17) Regular review of individual practice/outcome data	*Yes*
(18) Privileges granted based on formal evaluation	*No*
(19) Privileges subject to formal regular review based on documented competence	*No*
(20) Standardized electronic endoscopic procedures	*Yes*
(21) Policies in place to ensure timeliness/completeness of procedure reporting	*Yes*
(22) Patient centered service	*Yes*
(23) Patient feedback and responsive action	*Yes*

**Table 4:** Content validity: content comparison GRS-C/GRS-UK.

Item	Percentage of statements in common (%)
(1) Consent process	11/13 (85)
(2) Safety	10/13 (77)
(3) Comfort	10/11 (91)
(4) Quality of the procedure	9/11 (82)
(5) Appropriateness	10/15 (67)
(6) Communicating results to referrer	7/11 (64)
(7) Quality of access and equity of provision	9/13 (69)
(8) Timeliness	13/14 (93)
(9) Booking responsiveness and flexibility	7/10 (70)
(10) Privacy and dignity	11/12 (92)
(11) Aftercare	15/16 (94)
(12) Ability to provide feedback to the service	7/10 (70)

**Table 5:** Construct validity: select GRS-C statements versus auditable outcomes.

GRS-C statement	6-month GRS-C outcome	Measureable auditable outcome *at the time of completion of third iteration of the GRS*
(1.1) There is a published patient information sheet	Yes	180/272 (66%; 95% CI 60; 72) of patients had received an information sheet^*∗*^
(3.4) Unacceptable comfort levels prompt a review during the procedure; this review includes the technique, sedation level, and indication for the procedure	Yes	Did you feel the doctor and nurse were attentive to make sure that you were comfortable during the colonoscopy? 269/272 (99%; 95% CI 97; 100) of patients responded “yes”^*∗*^
(7.5) Facility and procedure information is available in written and/or electronic form in the most prevalent community languages, as determined by needs assessment	Yes	Was the information related to your colonoscopy provided in a language you could understand? 267/272 (98%; 95% CI 96; 99) responded yes^*∗*^
(8.5) Wait for urgent procedures is less than two weeks from referral	Yes	Wait list data at site confirms that patients are scoped within 2 weeks
(9.9) Patients are given a choice about the date and time of day of their appointment	Yes	Were you offered a choice of dates/times for your colonoscopy? 147/272 (54%; 95% CI 48; 60) responded “yes”^*∗*^

^**∗**^Data from patient satisfaction survey.

**Table 6:** Listed action plans.

Proposed action plan (site)	Complete
Patient information pamphlet (1)	Yes
Implement comfort monitoring score (1)	No
Increase frequency of committee review of quality indicators to twice yearly (1)	Yes
Increase frequency of endoscopist feedback to twice yearly (1)	Yes
Implement annual appropriateness audits and communicate it to endoscopists (1)	Yes
Rereview direct to procedure guidelines yearly (1, 3)	No (site 1) Yes (site3)
Implement policy for ensuring that pathology results are communicated to patient by endoscopist (1)	No
Translate facility and procedure information to an additional prevalent community language (1, 2)	No (site 1) Yes (site 2)
Include equality of access question on existing patient survey (1)	No
Increase frequency of communication of wait times to endoscopy team (1)	No
Add contact number to patient discharge sheet (1)	No
Make information concerning biopsies and follow-up mandatory field on report	No
Designate an “adverse events review committee” (1)	Yes
Create and distribute yearly patient survey (2, 3)	Yes
Implement fax feature of electronic reporting to have reports sent directly to referring physician (3)	Yes
Admin assistants to track cancellation rates (2)	Yes
Front desk to notify referring physician when an appointment is missed (2)	Yes
Secure a locked space for patients to keep belongings (2)	No
Internal memo to remind endoscopists to send pathology reports to referring physicians (3)	No
